# Dosimetric comparison of intensity modulated radiotherapy and three-dimensional conformal radiotherapy in patients with gynecologic malignancies: a systematic review and meta-analysis

**DOI:** 10.1186/1748-717X-7-197

**Published:** 2012-11-23

**Authors:** Baojuan Yang, Lin Zhu, Haiyan Cheng, Qi Li, Yunyan Zhang, Yashuang Zhao

**Affiliations:** 1 Department of Gynecologic Oncology, Cancer Hospital of Harbin Medical University, 150 Haping Street, Nangang District, Harbin, Heilongjiang Province, P. R. China; 2 Department of Epidemiology, Public Health College, Harbin Medical University, 157 Baojian Street, Nangang District, Harbin, Heilongjiang Province, P. R. China

**Keywords:** IMRT, 3D-CRT, DVH, Gynecologic malignancies, Meta-analysis

## Abstract

**Background:**

To quantitatively evaluate the safety and related-toxicities of intensity modulated radiotherapy (IMRT) dose–volume histograms (DVHs), as compared to the conventional three-dimensional conformal radiotherapy (3D-CRT), in gynecologic malignancy patients by systematic review of the related publications and meta-analysis.

**Methods:**

Relevant articles were retrieved from the PubMed, Embase, and Cochrane Library databases up to August 2011. Two independent reviewers assessed the included studies and extracted data. Pooled average percent irradiated volumes of adjacent non-cancerous tissues were calculated and compared between IMRT and 3D-CRT for a range of common radiation doses (5-45Gy).

**Results:**

In total, 13 articles comprised of 222 IMRT-treated and 233 3D-CRT-treated patients were included. For rectum receiving doses ≥30 Gy, the IMRT pooled average irradiated volumes were less than those from 3D-CRT by 26.40% (30 Gy, *p =* 0.004), 27.00% (35 Gy, *p* = 0.040), 37.30% (40 Gy, *p* = 0.006), and 39.50% (45 Gy, *p =* 0.002). Reduction in irradiated small bowel was also observed for IMRT-delivered 40 Gy and 45 Gy (by 17.80% (*p* = 0.043) and 17.30% (*p* = 0.012), respectively), as compared with 3D-CRT. However, there were no significant differences in the IMRT and 3D-CRT pooled average percent volumes of irradiated small bowel or rectum from lower doses, or in the bladder or bone marrow from any of the doses. IMRT-treated patients did not experience more severe acute or chronic toxicities than 3D-CRT-treated patients.

**Conclusions:**

IMRT-delivered high radiation dose produced significantly less average percent volumes of irradiated rectum and small bowel than 3D-CRT, but did not differentially affect the average percent volumes in the bladder and bone marrow.

## Background

Radiotherapy (RT) plays an important role in the adjuvant treatment of gynecologic malignancies, particularly in cervical and endometrial cancer. While RT has greatly improved local regional control of primary tumors [1-3], it has come at the cost of significant toxic effects to adjacent non-cancerous tissues [4,5]. In the late 1990s, the technique of three-dimensional conformal radiation therapy (3D-CRT) emerged as a preferred treatment for gynecologic malignancies, since it gave better target coverage and significantly reduced the radiation exposure to the bladder [6]. However, this technique did not appreciably reduce the amount of radiation exposure to the intestine or rectum [7]. More recent advances in computer technology have led to improvements on the 3D-CRT technique; one, in particular, being the development of intensity modulated radiation therapy (IMRT) [8-12]. In contrast to 3D-CRT, which uses uniform fields, IMRT generates non-uniform fields to achieve better planning target volume coverage, while decreasing unnecessary radiation exposure to normal organs [9,13,14]. Therefore, IMRT has become a common strategy for whole pelvic radiotherapy (WPRT), and has been shown to offer more accurate dose distributions and tighter dose gradients to targets and to reduce toxic risk and undesirable side effects to the rectum, bladder, small bowel, and pelvic bones [15-18].

IMRT has also proven an efficacious and safe method of treating head, neck, lung, central nervous system, breast, and prostate cancers [19-23]. While the method has been applied to cervical and endometrial cancers as well [17,18,24-31], the reported findings on its utility and safety in these patients have been controversial. Thus, in the late 2000s, the National Comprehensive Cancer Network (NCCN) reported that IMRT treatment for gynecologic malignancy was not sufficiently well-established for general recommendation [32]. The main problems cited were the facts that the target site and parameters of posture immobilization remained to be precisely defined, and that the repeatability of an IMRT model remained to be demonstrated [32].

Nonetheless, the previous successes of IMRT in other cancer patients have promoted significant research interest to evaluate its promise for treating gynecologic malignancy patients [33]. In addition, the proven benefits of IMRT over the 3D-CRT technique have led to several studies to determine whether IMRT is superior to 3D-CRT for the clinical treatment of gynecologic malignancies. With the aim of resolving the inconsistencies that have arisen from these studies, we conducted a systematic review and meta-analysis of IMRT and 3D-CRT use in gynecologic malignancy patients. In addition to quantitatively evaluating the safety of IMRT in these patients, we also performed a comparative analysis of the dose–volume histograms (DVHs) generated for both IMRT and 3D-CRT. Finally, the acute and chronic toxicity effects of IMRT and 3D-CRT are systematically reviewed.

## Materials and methods

### Primary search strategy

The PubMed, Embase, and Cochrane Library databases were searched for relevant publications by using the following keywords: “radiotherapy, intensity modulated”, “IMRT”, “cervical cancer”, “cervix cancer”, “cervical carcinoma”, “cervix carcinoma”, “endometrial cancer”, “endometrial carcinoma”, and “gynecologic malignancies”. The upper publication date was August 2011 and no lower date was set. These terms were then combined with the search terms for the following study designs: “practice guideline”, “systematic review”, “meta-analysis”, and “review”. In addition, the reference lists of all pertinent articles found in PubMed were manually searched.

The Physician Data Query (PDQ) clinical trials database and the proceedings of the 1980–2010 annual meetings of the American Society of Clinical Oncology (ASCO) and the American Society of Radiation Therapist (ASTRO) were also searched for reports of new or on-going trials.

### Criteria for study inclusion and exclusion

**Figure 1:**
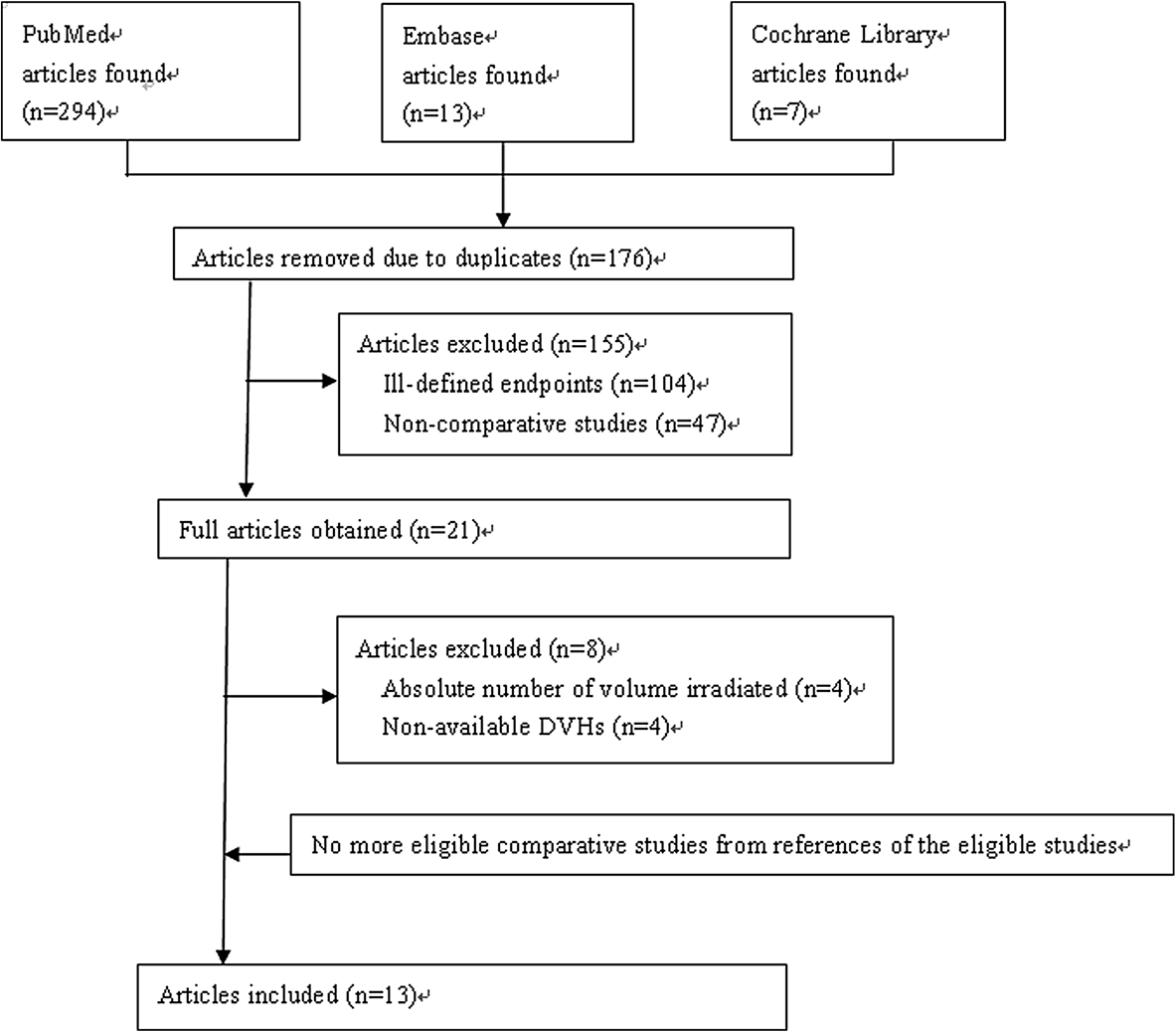
Flow chart of identification and selection of papers for study.

A study was selected for inclusion if it provided information on DVHs of different irradiated organs that had been treated with IMRT or 3D-CRT. Those studies were then selected for the following criteria: 1) prescription dose of Gy or 50.4 Gy, for either IMRT or 3D-CRT, with all patients having received radiation doses of 1.8 Gy/day; 2) irradiated normal organs at risk being small bowel, bladder, rectum, and bone marrow; 3) data from the DVHs in irradiated organs at risk being relative number and not the actual measured value; and 4) studies being independent and not replicates of a single population. If studies were found to overlap, the largest dataset was selected for inclusion. A flowchart of the strategy used for this systematic review of the literature is presented in Figure 1.

### Data extraction

The data from each included study were extracted by two reviewers (Lin Zhu and Baojuan Yang), who worked independently and used a standardized form for data collection. Any subsequent discrepancies were discussed and revised until a consensus was achieved. Information extracted from each article included the first author, country of origin, number of patients, normal organs irradiated, prescribed dose of IMRT and 3D-CRT, and average percent irradiated volumes of the organs at risk (OARs) at various radiation doses (from 5 Gy to 45 Gy, the interval of each level was 5 Gy) in DVHs.

In regards to the DVHs information, if the authors did not list the average percent irradiated volumes of OARs, we measured it based on the figures in the article. If the prescribed dose in the study was 50.4 Gy (indicating patients had received an additional three days of 1.8 Gy/day), we only extracted the data for radiated doses <45 Gy.

### Statistical analysis

To assess heterogeneity of the studies, a fixed effect model based on the Mantel-Haenszel method was used to calculate the pooled average percent irradiated volumes, if *I*^*2*^ was less than 50%; otherwise, a random effect model was used. The pooled average percent irradiated volumes for IMRT and 3D-CRT treatment plans were compared at each radiation dose in OARs (including small bowel, bladder, rectum, and bone marrow). Differences were considered to be statistically significant if the *p*-value was less than 0.05. Publication bias was evaluated by funnel plot, followed by a quantitative analysis using a linear regression approach and rank correlation method [34,35]. For this study, all data analyses were performed by the Comprehensive Meta-analysis software (version 2; Biostat, Inc., USA), and the statistical analyses were carried out with SAS software (version 9.1; SAS Institute, USA).

## Results

### Characteristics of the included studies

In total, 13 articles, which compared the extents of DVHs in IMRT and 3D-CRT for endometrial and cervical cancer patients, were included. Ten of these reported on the irradiated volumes of small bowel, nine on rectum, eight on bladder, and six on bone marrow. The 13 articles comprised a total of 222 patients treated with IMRT and 233 patients treated with 3D-CRT. The prescribed dose was either 45 Gy or 50.4 Gy, and all patients received treatment at 1.8 Gy/y. The characteristics of the included articles are summarized in Table 1.

**Table 1:** Basic characteristics of papers analyzed

**First author, [Reference]**	**Country**	**Prescribed dose, Gy**	**Sample size**		**Organs at risk**	**Level of the dose, Gy**
			IMRT^*^	**3D-CRT**^**+**^		
Heron DE [26]	USA	45	10	10	Rectum, Small bowel, Bladder	10, 20, 30, 40, 45
Chen MF [36]	Taiwan	50.4	33	35	Rectum, Small bowel, Bladder, Bone marrow	5, 10, 15, 20, 25, 30, 35, 40, 45
Mell LK [30]	USA	45	7	7	Rectum, Small bowel, Bladder, Bone marrow	5, 10, 20, 30, 40, 45
Igdem S [31]	Turkey	45 or 50.4	10	10	Rectum, Small bowel, Bladder, Bone marrow	5, 10, 15, 20, 25, 30, 40, 45
Roeske JC [37]	USA	45	10	10	Rectum, Small bowel, Bladder	5, 10, 15, 20, 25, 30, 35, 40, 45
Portelance L [17]	USA	45	10	10	Rectum, Small bowel, Bladder	45
Lujan AE [38]	USA	45	10	10	Bone marrow	5, 10, 15, 20, 25, 30, 35, 40, 45
Brixey CJ [39]	USA	45	36	88	Iliac crest, Lumbar spine, Sacrum	5, 10, 15, 20, 25, 30, 35, 40, 45
Ahmed RS [27]	USA	45	5	5	Bone marrow	5, 10, 15, 20, 25, 30, 35, 40, 45
Mell LK [37]	USA	45	37	0	Bone marrow	10, 20, 30, 40
Mundt AJ [38]	USA	45	36	30	Small bowel	5, 10, 15, 20, 25, 30, 35, 40, 45
Salama JK [40]	USA	45	13	13	Rectum, Small bowel	5, 10, 15, 20, 25, 30, 35, 40, 45
Georg D [41]	Austria	50.4	5	5	Rectum, Small bowel, Bladder	5, 10, 15, 20, 25, 30, 35, 40, 45

^*^ intensity modulated radiotherapy; ^+^ three-dimensional conformal radiotherapy.

### Pooled average percent irradiated volumes of IMRT and 3D-CRT

The pooled average percent irradiated volumes of IMRT and 3D-CRT were calculated for different OARs and compared for each irradiated level (Table 2). For rectums treated with <30 Gy, the pooled average irradiated volumes were not statistically different between IMRT and 3D-CRT. However, rectums that received ≥30 Gy doses had significantly lower pooled average irradiated volumes for IMRT (30 Gy, 68.00 (95% CI: 56.60-77.50); 35 Gy, 61.80 (95% CI: 48.80-73.30); 40 Gy, 48.10 (95% CI: 26.90-70.10); and 45 Gy, 31.30 (95% CI: 13.20-57.70)) than for 3D-CRT (94.40 (95% CI: 86.20-96.80); 88.80 (95% CI: 74.70-95.50); 85.40 (95% CI: 74.40-92.20); and 70.80 (95% CI: 59.60-80.00)). Thus, the pooled average irradiated volumes of IMRT were lower than that of 3D-CRT by 26.40% (*p* = 0.004), 27.00% (*p* = 0.040), 37.30% (*p* = 0.006), and 39.50% (*p* = 0.002), respectively. Furthermore, a statistically significant dose–response relationship was observed between increasing Gy of irradiation doses and decreasing pooled average percent volumes (*P* = 0.003).

**Table 2:** Pooled-average volumes irradiated (%) of IMRT and 3D-CRT in different risk organs and the publication bias

**Organs**	**Irradiated dose, Gy**	**No. of studies**	**No. of cases**	**Pooled volume irradiated, % (95% CI)**		**Egger’s *p-*value**		**Begg’s *p-*value**		**Studies trimmed**		**Adjusted volume irradiated, %**		***p-*value**^**⋆**^
				IMRT^*^	**3D-CRT**^**+**^	IMRT^*^	**3D-CRT**^**+**^	IMRT^*^	**3D-CRT**^**+**^	IMRT^*^	**3D-CRT**^**+**^	IMRT^*^	**3D-CRT**^**+**^	
Rectum	10	7	93	93.10	96.80	0.015	0.211	1.000	0.881	4	N/A	87.70	N/A	0.308
				(83.80-97.30)	(90.00-99.00)									
	20	7	93	87.80	97.50	0.303	0.406	0.327	0.624	N/A	N/A	N/A	N/A	0.077
				(73.70-94.90)	(90.50-99.40)									
	25	5	76	90.00	97.40	0.045	0.142	0.368	0.652	3	N/A	86.00	N/A	0.101
				(79.90-95.30)	(89.30-99.40)									
	30	7	93	68.00	94.40	0.108	0.437	0.086	0.807	N/A	N/A	N/A	N/A	0.004
				(56.60-77.50)	(86.20-96.80)									
	35	4	66	61.80	88.80	0.100	0.071	0.071	0.051	2	N/A	55.00	N/A	0.040
				(48.80-73.30)	(74.70-95.50)									
	40	7	93	48.10	85.40	0.030	0.052	0.174	0.308	N/A	3	N/A	80.10	0.002
				(26.90-70.10)	(74.40-92.20)									
	45	8	111	31.30	70.80	0.462	0.001	0.368	0.024	N/A	3	N/A	62.10	0.006
				(13.20-57.70)	(59.60-80.00)									
Small bowel	5	5	102	97.20	96.70	0.001	0.271	0.076	1.000	1	N/A	96.70	N/A	0.831
				(90.90-99.20)	(89.50-99.00)									
	10	8	129	88.60	90.00	0.004	0.020	0.226	0.009	4	3	82.50	86.20	0.795
				(79.00-94.20)	(81.30-95.00)									
	15	6	112	85.90	86.10	0.011	0.001	0.060	0.060	2	1	80.30	83.80	0.985
				(74.40-92.70)	(75.00-92.80)									
	20	8	129	72.30	78.20	0.637	0.001	0.711	0.009	N/A	3	N/A	71.80	0.319
				(63.70-83.70)	(66.90-86.50)									
	25	6	112	62.20	73.90	0.036	0.114	1.000	0.060	3	N/A	77.40	N/A	0.431
				(44.00-77.50)	(49.10-89.30)									
	30	8	129	43.70	54.80	0.133	0.062	0.216	0.108	N/A	N/A	N/A	N/A	0.186
				(35.00-52.80)	(34.30-73.80)									
	35	5	102	36.60	50.60	0.076	0.002	0.050	0.027	N/A	0	N/A	60.80	0.137
				(27.70-46.60)	(27.50-73.40)									
	40	8	129	24.70	42.50	0.049	0.005	0.266	0.035	3	2	28.10	58.10	0.043
				(17.30-34.10)	(24.00-63.40)									
	45	9	147	18.60	35.90	0.008	0.000	0.076	0.001	4	4	20.70	55.80	0.012
				(12.60-26.70)	(21.90-52.80)									
Bladder	10	6	80	95.50	96.00	0.334	0.001	0.851	0.133	N/A	2	N/A	96.60	0.871
				(86.40-98.60)	(88.40-98.70)									
	20	6	80	88.90	96.00	0.151	0.001	0.707	0.133	N/A	2	N/A	96.60	0.169
				(73.20-95.90)	(88.40-98.70)									
	25	4	63	90.90	96.60	0.015		0.089	0.497	2	N/A	86.00	N/A	0.258
				(76.60-96.80)	(87.30-99.10)									
	30	6	80	81.20	94.60	0.227	0.153	0.133	0.260	N/A	N/A	N/A	N/A	0.079
				(61.60-92.10)	(83.80-98.40)									
	35	3	53	80.60	89.70	0.936	0.029	1.000	0.296	N/A	2	N/A	85.00	0.396
				(57.70-92.70)	(70.70-96.90)									
	40	6	80	53.60	76.70	0.260	0.176	0.133	0.133	N/A	N/A	N/A	N/A	0.090
				(33.30-72.80)	(59.10-88.30)									
	45	7	98	50.10	79.30	0.059	0.005	0.071	0.133	N/A	2	N/A	64.70	0.062
				(31.70-68.50)	(54.10-92.60)									
Bone marrow	5	5	91/143	95.20	97.90	0.833	0.112	1.000	0.807	N/A	N/A	N/A	N/A	0.501
				(86.10-98.40)	(83.60-99.80)									
	10	6	101/153	87.30	95.50	0.138	0.276	0.230	0.133	N/A	N/A	N/A	N/A	0.129
				(73.20-93.60)	(86.00-98.60)									
	15	5	94/146	74.10	90.80	0.005	0.039	0.624	0.086	3	1	68.30	90.50	0.191
				(61.10-83.90)	(80.60-95.90)									
	20	6	101/153	67.40	68.50	0.165	0.005	0.368	0.707	N/A	3	N/A	63.40	0.301
				(47.50-82.50)	(55.40-79.30)									
	25	4	61/113	62.30	83.40	0.319	0.097	1.000	0.089	N/A	N/A	N/A	N/A	0.061
				(45.20-76.80)	(66.50-92.70)									
	30	5	68/120	47.10	59.40	0.170	0.004	0.060	0.027	N/A	1	N/A	61.20	0.261
				(32.50-62.20)	(44.00-73.50)									
	35	3	51/103	36.80	51.70	0.065	0.074	0.296	1.000	N/A	N/A	N/A	N/A	0.307
				(19.90-57.80)	(32.60-70.40)									
	40	5	68/120	29.70	40.20	0.859	0.187	1.000	0.462	N/A	N/A	N/A	N/A	0.327
				(17.50-45.70)	(26.50-55.70)									
	45	4	61/113	12.80	31.00	0.070	0.099	0.308	0.089	N/A	N/A	N/A	N/A	0.068
				(5.00-28.90)	(18.00-47.90)									

^*^ intensity modulated radiotherapy; ^+^ three-dimensional conformal radiotherapy; ^**⋆**^*p-*value for comparison difference of pooled volume irradiated between IMRT and 3D-CRT.

In small bowel, the pooled average percent volumes were significantly lower (by 17.80%) for IMRT than for 3D-CRT at a radiation dose of 40 Gy (IMRT: 24.70% (95% CI: 17.30-34.10) and 3D-CRT: 42.50% (95% CI: 24.00-63.40); *p = 0*.043). Similarly, at a dose of 45 Gy, the pooled average percent volumes were 17.30% lower in IMRT (IMRT: 18.60% (95% CI: 12.60-26.70) and 3D-CRT: 35.90% (95% CI: 21.90-52.80); *p* = 0.012). At low doses (<20 Gy), the pooled average percent volumes of small bowel irradiated with IMRT were similar to those for patients who received 3D-CRT treatment (*p >* 0.05). Likewise, the doses between 25 Gy and 35 Gy did not produce significantly different effects (*p >* 0.05), but irradiation with IMRT did yield > 10% less percent pooled average percent volumes than 3D-CRT.

The results of bladder and bone marrow from our meta-analysis revealed that the pooled average irradiated volumes in IMRT were lower than those in 3D-CRT. Although the differences were more obvious for the higher doses of irradiation, none reached statistical significance (Table 2).

### Publication bias

**Figure 2:**
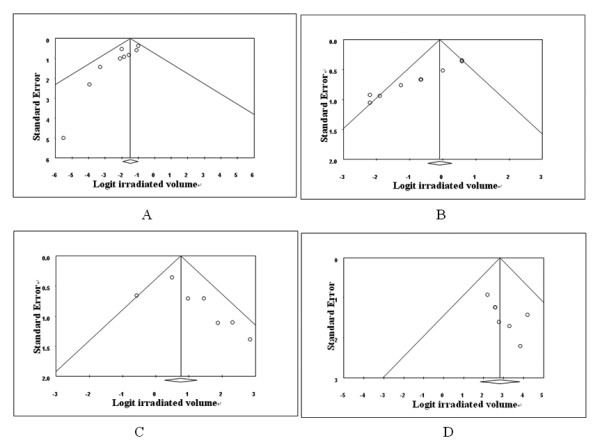
**Funnel plots for pooled average percent irradiated volume.A**. Small bowel at 45Gy with IMRT. **B**. Small bowel at 45Gy with 3D-CRT. **C**. Rectum at 30Gy with IMRT. **D**. Rectum at 30Gy with 3D-CRT.

The graphical funnel plots of pooled average percentage volumes of small bowel irradiated at 45 Gy and rectum irradiated at 30 Gy by IMRT and 3D-CRT are shown in Figure 2. Although the dots were not entirely localized to the bottom of the inverted funnel plots, they were distributed symmetrically around the central axis. Using the Begg’s rank correlation method and Egger’s linear regression approach, we identified publication bias for the 40 Gy and 45 Gy radiation dose of the rectum with 3D-CRT and for the 10 Gy, 25 Gy, and 40 Gy radiation doses of the rectum with IMRT. Publication bias was also found in several radiation dose levels of small bowel with both IMRT and 3D-CRT, with the exceptions of 20 Gy, 30 Gy, and 35 Gy with IMRT and 5 Gy, 25 Gy, and 30 Gy with 3D-CRT. The results for the bladder and bone marrow very nearly indicated publication bias for all radiation doses, except for 25 Gy for the bladder and 15 Gy for bone marrow. The detailed results are presented in Table 2.

### Acute and chronic toxicities

The acute and chronic toxicity effects of IMRT and 3D-CRT were evaluated by investigating the reported side effects of gastrointestinal (GI), genitourinary (GU), and hematologic toxicity for each. Five studies that reported on the toxic effects of IMRT and 3D-CRT were identified, including four on acute toxicity [25,36,42,43] and three on chronic toxicity [36,39,43]. The acute (Table 3) and chronic (Table 4) toxicities reported in recent series of adjuvant IMRT for gynecologic malignancies were graded using the Radiation Therapy Oncology Group scales. Specifically, the worst toxicity was noted and graded according to the following 4-point scale: 0, none; 1, mild, no medications required; 2, moderate, medications required; and 3–4, severe, treatment breaks, hospitalization required.

**Table 3:** Acute toxicities of IMRT and 3D-CRT

**Reference**	**Grade**	**Cases of GI**^**++**^		**Cases of GU**^**#**^		**Cases of Hematology**	
		IMRT^*^	**3D-CRT**^**+**^	IMRT^*^	**3D-CRT**^**+**^	IMRT^*^	**3D-CRT**^**+**^
Mundt *et al.*[25]	0	5	0	28	21	N/D	N/D
	1	11	3	8	7	N/D	N/D
	2	24	32	4	7	N/D	N/D
	3-4	0	0	0	0	N/D	N/D
	total	40	35	40	35	N/D	N/D
Beriwal *et al.*[42]	0	9	N/D	12	N/D	6	N/D
	1	4	N/D	16	N/D	7	N/D
	2	22	N/D	7	N/D	13	N/D
	3-4	1	N/D	1	N/D	10	N/D
	total	36	N/D	36	N/D	36	N/D
Hasselle *et al.*[43]	0	28	N/D	68	N/D	N/D	N/D
	1	31	N/D	27	N/D	N/D	N/D
	2	50	N/D	16	N/D	N/D	N/D
	3-4	2	N/D	0	N/D	N/D	N/D
	total	111	N/D	111	N/D	N/D	N/D
Chen *et al.*[36]	0	21	7	23	14	14	16
	1	4	8	6	12	8	5
	2	8	20	4	9	9	11
	3-4	0	0	0	0	2	3
	total	33	35	33	35	33	35

^*^ intensity modulated radiotherapy; ^+^ three-dimensional conformal radiotherapy^; ++^ gastrointestinal; ^#^ genitourinary.

**Table 4:** Chronic toxicities of IMRT and 3D-CRT

**Reference**	**Grade**	**Cases of GI**^**++**^		**Cases of GU**^**#**^	
		IMRT^*^	**3D-CRT**^**+**^	IMRT^*^	**3D-CRT**^**+**^
Mundt *et al.*[39]	0	32	15	N/D	N/D
	1	3	9	N/D	N/D
	2	1	5	N/D	N/D
	3-4	0	1	N/D	N/D
	total	36	30	N/D	N/D
Hasselle *et al.*[43]	0	81	N/D	91	N/D
	1	15	N/D	13	N/D
	2	11	N/D	2	N/D
	3-4	4	N/D	5	N/D
	total	111	N/D	111	N/D
Chen *et al.*[36]	0	31	23	30	27
	1	2	4	2	5
	2	0	6	0	2
	3-4	0	2	1	1
	total	33	35	33	35

^*^ intensity modulated radiotherapy; ^+^ three-dimensional conformal radiotherapy;

^++^ gastrointestinal; ^#^ genitourinary.

In the studies by Mundt *et al.*[25] and Chen *et al.*[36], none of the reported toxicities of gynecologic patients treated with either IMRT or 3D-CRT met the criteria of severe acute GI or GU toxicity, but IMRT treatment did result in fewer patients with moderate toxicity and needing medications than did 3D-CRT. In the studies by Beriwal *et al.*[42] and Hasselle *et al.*[43], severe acute GI, GU, and hematologic toxicities were found in patients who underwent IMRT treatment. However, these studies only evaluated patients with IMRT and did not consider 3D-CRT treatment.

For chronic toxicities, most of the patients receiving IMRT had no or mild side effects of GI and GU. The incidence of severe GI side effects with IMRT was 2.20% (4/180), and with 3D-CRT, the rate reached up to 4.60% (3/65). Although Hasselle *et al.*[43] concluded that IMRT caused a severe GU side effect, they did not compare it with the incidence in 3D-CRT treatment groups. In the study by Chen *et al.*[36], one patient from each treatment group (3D-CRT: 1/35; IMRT: 1/36) was reported to have experienced such severe side effects from the treatment that hospitalization was required and treatment terminated.

## Discussion

Because IMRT can deliver treatment to target organs while reducing the volumes of proximal normal structures that are irradiated, it appears to offer several advantages over conventional techniques for the treatment of malignancies [14] . However, studies on the effects of IMRT in reducing the irradiated volumes of the rectum, small bowel, bladder, and bone marrow have reported inconsistent findings [18,26,27,30,31,36-41,44,45]. Therefore, we carried out a systematic review to identify all the relevant studies presenting data on DVHs of IMRT and 3D-CRT. Consequently, data from 13 studies were analyzed by calculating the pooled average percent irradiated volumes, and they were used in a comparative analysis of the effects of IMRT and 3D-CRT in the rectum, small bowel, bladder, and bone marrow at various radiation doses.

The studies by Heron *et al.*[26], Igdem *et al.*[31], and Roeske *et al.*[37] reported that IMRT at doses of 30 Gy, 40 Gy, and 45 Gy significantly reduced the irradiated volume of the rectum, as compared to 3D-CRT. Chen *et al.*[36] reported that, when patients received 70% of the prescribed dose with IMRT, the average percent volume of irradiated rectum was significantly less (*p* < 0.05). However, the study by Mell *et al.*[30] found no significant reduction in average percent volumes irradiated by IMRT at those same doses. Our meta-analysis indicated that the pooled average percent volumes of irradiated rectum (at doses of 30 Gy, 35 Gy, 40 Gy, and 4 5Gy) were significantly lower in IMRT than in 3D-CRT. Moreover, this reduction manifested a dose response relationship with increasing radiation doses (*P* = 0.003). Since some publication bias existed in our meta-analysis, we adjusted the reduction of volumes irradiated by using the trim and fill method, and we found that the differences retained statistical significance. This result illustrated that, the higher the radiation dose prescribed, the better IMRT was at reducing the average percent irradiated volumes for the rectum, as compared to 3D-CRT.

Some studies have reported that IMRT treatment of gynecologic malignancies more effectively protects the small bowel compared to the 3D-CRT technique, especially when radiation doses <20 Gy are used [31]. In the studies by Heron *et al.*[26] and Roeske *et al.*[37], it was found that IMRT-delivered doses of >30 Gy and >45 Gy, respectively, produced remarkably less average irradiated volumes of OARs (by more than 10-fold) than 3D-CRT. Other studies also reported that >25 Gy doses delivered by IMRT were more beneficial than those delivered by 3D-CRT [39,44] . Our meta-analysis results showed that after weighing the sample sizes, IMRT at 40 Gy and 45 Gy significantly reduced the pooled average percent irradiated volumes of the small bowel by 17.80% (*p* = 0.043) and 17.30% (*p* = 0.012), respectively. However, at 35 Gy and below, no statistically significant reduction was found between IMRT and 3D-CRT in the pooled average percent of irradiated volumes. In this meta-analysis, there was no publication bias detected for data related to IMRT-delivered 20 Gy, 30 Gy, or 35 Gy or for 3D-CRT-delivered 5 Gy, 20 Gy, or 25 Gy. Although publication bias was observed for data related to both IMRT- and 3D-CRT-delivered 40 Gy and 45 Gy radiation doses, the reduction of pooled average percent irradiated volumes remained significant after adjusting with the trim and fill method.

This meta-analysis found no statistically significant evidence to support the theory that IMRT was an effective approach to reduce the irradiated volumes of the bladder.

Considering the effects of IMRT and 3D-CRT on bone marrow, Brixey *et al.*[40] showed that IMRT produced no obvious reduction in the volumes of OARs irradiated at the 10 Gy and 30 Gy doses, but reported a statistically significant reduction for doses of 20 Gy, 40 Gy, and 45 Gy (*p* < 0.001). In the studies by Lujun *et al.*[38] and Ahmed *et al.*[27], the average percent volumes of irradiated OARs were found to be reduced at several high radiation levels delivered by IMRT. In contrast, Chen *et al.*[36]demonstrated a significant reduction in the volume of irradiated bone marrow when IMRT delivered doses of 20 Gy and below. When these seemingly inconsistent results were combined in our meta-analysis, IMRT was found to reduce the average percent volumes of irradiated bone marrow at all radiation doses, but the findings did not reach statistical significance (*p* > 0.05). Publication bias was observed only for the IMRT-delivered dose of 15 Gy and 3D-CRT-delivered doses of 15 Gy, 20 Gy, and 30 Gy. After adjusting the reduction of irradiated volumes by using the trim and fill method, there was still no statistically significant reduction found between IMRT and 3D-CRT.

High heterogeneity was found for the data of bladder and rectum irradiation from high radiation doses delivered by IMRT and for the data of small bowel and bladder irradiation from 40 Gy and 45 Gy doses delivered by 3D-CRT. Potential explanations exist to explain these instances of heterogeneity. First, the OARs in the abdominal cavity are not static and are in continual motion, and the volumes of irradiated organs are known to be impacted by different postures. Second, the RT physicians defined the extent of OARs that were reported in each study, and they may not have abided by a unified standard. Third, the data from each study was generated independently and may have been influenced by the particular study design.

In our meta-analysis, we determined that toxicity occurred with significantly lower frequency in the IMRT treated patients than in the 3D-CRT patients [25,39,46]. In the studies by Mundt *et al.*[25] and Chen *et al.*[36], adjuvant IMRT was reported to be well-tolerated with low incidences of acute and chronic toxicity, as compared with 3D-CRT. Although several patients in the studies by Beriwal *et al.*[42] and Hasselle *et al.*[43] suffered severe acute and chronic toxicities from IMRT, the incidence of these side effects was not compared with that of 3D-CRT. These two research studies prompted us to theorize that the most significant factor correlated to IMRT-induced toxicity in gynecologic patients is the organ volume receiving 100% (45 Gy) of the prescription dose [47]. Likewise, Rose *et al.*[48] provided evidence that hematologic toxicity increased with increasing volumes of irradiated pelvic bone marrow.

Finally, the collected IMRT dosimetric data from gynecologic patients used in our meta-analysis suggested that IMRT is safe for use as a treatment of gynecologic cancers. However, all of the research studies with which our meta-analysis was carried out were observational. It is generally believed that findings from observational studies are not as accurate as those from randomized controlled trials, since they can easily overestimate the magnitude of effects. Another limitation in our study was the small sample size and uneven quality of the samples. Thus, our conclusions need to be validated by larger samples and more studies to confirm the benefits of IMRT in patients with gynecologic malignancy and to further study the different acute and chronic toxicities produced by IMRT and 3D-CRT.

## Conclusions

This study suggested that IMRT significantly reduced the average percent irradiated volume of the rectum resulting from >30 Gy doses and of the small bowel from 45 Gy. Furthermore, in the bladder and bone marrow, the advantages of IMRT over 3D-CRT were not significant for any of the radiation doses examined.

## Abbreviations

IMRT: Intensity modulated radiotherapy; DVHs: Dose–volume histograms; 3D-CRT: Three-dimensional conformal radiotherapy; RT: Radiotherapy; WPRT: Whole pelvic radiotherapy; NCCN: National comprehensive cancer network; PDQ: Physician data query; ASCO: The American society of clinical oncology; ASTRO: American society of radiation therapist; OAR: Organ at risk.

## Competing interests

The author(s) declare that they have no competing interests.

## Authors’ contributions

BY carried out the molecular genetic studies, participated in the sequence alignment and drafted the manuscript. LZ carried out the immunoassays. HC participated in the sequence alignment. QL participated in the design of the study and performed the statistical analysis. YZ and YZ conceived of the study, and participated in its design and coordination and helped to draft the manuscript. All authors read and approved the final manuscript.

## References

[B1] Rose PG Bundy BN Watkins EB Thigpen JT Deppe G Maiman MA Clarke-Pearson DL Insalaco S Concurrent cisplatin-based radiotherapy and chemotherapy for locally advanced cervical cancer N Engl J Med 1999 340 1144 1153 10.1056/NEJM199904153401502 10202165

[B2] Creutzberg CL van Putten WL Koper PC Lybeert ML Jobsen JJ Warlam-Rodenhuis CC De Winter KA Lutgens LC van den Bergh AC van de Steen-Banasik E Beerman H van Lent M Surgery and postoperative radiotherapy versus surgery alone for patients with stage-1 endometrial carcinoma: multicentre randomised trial. PORTEC Study Group. Post Operative Radiation Therapy in Endometrial Carcinoma Lancet 2000 355 1404 1411 10.1016/S0140-6736(00)02139-5 10791524

[B3] Papp Z Csapo Z Mayer A Hupuczi P [Wertheim-operation: 5-year survival of 501 consecutive patients with cervical cancer] Orv Hetil 2006 147 537 545 16696377

[B4] Yeoh E Radiotherapy: long-term effects on gastrointestinal function Curr Opin Support Palliat Care 2008 2 40 44 10.1097/SPC.0b013e3282f4451f 18685393

[B5] Vargas C Martinez A Kestin LL Yan D Grills I Brabbins DS Lockman DM Liang J Gustafson GS Chen PY Vicini FA Wong JW Dose-volume analysis of predictors for chronic rectal toxicity after treatment of prostate cancer with adaptive image-guided radiotherapy Int J Radiat Oncol Biol Phys 2005 62 1297 1308 10.1016/j.ijrobp.2004.12.052 16029785

[B6] Gerstner N Wachter S Knocke TH Fellner C Wambersie A Potter R The benefit of Beam's eye view based 3D treatment planning for cervical cancer Radiother Oncol 1999 51 71 78 10.1016/S0167-8140(99)00038-9 10386719

[B7] Barillot I [Cervix carcinomas: place of intensity-modulated radiotherapy] Cancer Radiother 2009 13 507 510 10.1016/j.canrad.2009.05.015 19695927

[B8] Purdy JA Intensity-modulated radiation therapy Int J Radiat Oncol Biol Phys 1996 35 845 846 10.1016/0360-3016(96)00223-4 8690655

[B9] Saw CB Ayyangar KM Enke CA MIMiC-based IMRT- part I Med Dosim 2001 26 1 11417500 10.1016/s0958-3947(01)00054-1

[B10] Saw CB Ayyangar KM Enke CA MLC-based IMRT-Part II Med Dosim 2001 26 111 112 10.1016/S0958-3947(01)00065-6 11444512

[B11] Bucci MK Bevan A Roach M 3rd Advances in radiation therapy: conventional to 3D, to IMRT, to 4D, and beyond CA Cancer J Clin 2005 55 117 134 10.3322/canjclin.55.2.117 15761080

[B12] Miles EA Clark CH Urbano MT Bidmead M Dearnaley DP Harrington KJ A'Hern R Nutting CM The impact of introducing intensity modulated radiotherapy into routine clinical practice Radiother Oncol 2005 77 241 246 10.1016/j.radonc.2005.10.011 16298002

[B13] Woo SY Sanders M Grant W Butler EB Does the "peacock" have anything to do with radiotherapy? Int J Radiat Oncol Biol Phys 1994 29 213 214 10.1016/0360-3016(94)90250-X 8175435

[B14] Whitton A Warde P Sharpe M Oliver TK Bak K Leszczynski K Etheridge S Fleming K Gutierrez E Favell L Green E Organisational standards for the delivery of intensity-modulated radiation therapy in Ontario Clin Oncol (R Coll Radiol) 2009 21 192 203 10.1016/j.clon.2008.10.005 19062263

[B15] Nutting CM Convery DJ Cosgrove VP Rowbottom C Padhani AR Webb S Dearnaley DP Reduction of small and large bowel irradiation using an optimized intensity-modulated pelvic radiotherapy technique in patients with prostate cancer Int J Radiat Oncol Biol Phys 2000 48 649 656 10.1016/S0360-3016(00)00653-2 11020560

[B16] Intensity-modulated radiotherapy current status and issues of interest Int J Radiat Oncol Biol Phys 2001 51 880 914 11704310 10.1016/s0360-3016(01)01749-7

[B17] Portelance L Chao KS Grigsby PW Bennet H Low D Intensity-modulated radiation therapy (IMRT) reduces small bowel, rectum, and bladder doses in patients with cervical cancer receiving pelvic and para-aortic irradiation Int J Radiat Oncol Biol Phys 2001 51 261 266 11516876 10.1016/s0360-3016(01)01664-9

[B18] Georg P Georg D Hillbrand M Kirisits C Potter R Factors influencing bowel sparing in intensity modulated whole pelvic radiotherapy for gynaecological malignancies Radiother Oncol 2006 80 19 26 10.1016/j.radonc.2006.04.014 16766068

[B19] Esik O Bortfeld T Bendl R Nemeth G Schlegel W Inverse radiotherapy planning for a concave-convex PTV in cervical and upper mediastinal regions. Simulation of radiotherapy using an Alderson-RANDO phantom. Planning target volume Strahlenther Onkol 1997 173 193 200 10.1007/BF03039288 9111607

[B20] Meeks SL Buatti JM Bova FJ Friedman WA Mendenhall WM Zlotecki RA Potential clinical efficacy of intensity-modulated conformal therapy Int J Radiat Oncol Biol Phys 1998 40 483 495 10.1016/S0360-3016(97)00819-5 9457839

[B21] Hong L Hunt M Chui C Spirou S Forster K Lee H Yahalom J Kutcher GJ McCormick B Intensity-modulated tangential beam irradiation of the intact breast Int J Radiat Oncol Biol Phys 1999 44 1155 1164 10.1016/S0360-3016(99)00132-7 10421550

[B22] Saarilahti K Kouri M Collan J Hamalainen T Atula T Joensuu H Tenhunen M Intensity modulated radiotherapy for head and neck cancer: evidence for preserved salivary gland function Radiother Oncol 2005 74 251 258 10.1016/j.radonc.2004.11.004 15763305

[B23] Vora SA Wong WW Schild SE Ezzell GA Halyard MY Analysis of biochemical control and prognostic factors in patients treated with either low-dose three-dimensional conformal radiation therapy or high-dose intensity-modulated radiotherapy for localized prostate cancer Int J Radiat Oncol Biol Phys 2007 68 1053 1058 10.1016/j.ijrobp.2007.01.043 17398023

[B24] Sirak I Kasaova L Petera J Vosmik M Zoul Z Intensity modulated radiation therapy technique in the treatment of gynecologic malignancies Ceska Gynekol 2010 75 177 181 20731296

[B25] Mundt AJ Lujan AE Rotmensch J Waggoner SE Yamada SD Fleming G Roeske JC Intensity-modulated whole pelvic radiotherapy in women with gynecologic malignancies Int J Radiat Oncol Biol Phys 2002 52 1330 1337 10.1016/S0360-3016(01)02785-7 11955746

[B26] Heron DE Gerszten K Selvaraj RN King GC Sonnik D Gallion H Comerci J Edwards RP Wu A Andrade RS Kalnicki S Conventional 3D conformal versus intensity-modulated radiotherapy for the adjuvant treatment of gynecologic malignancies: a comparative dosimetric study of dose-volume histograms small star, filled Gynecol Oncol 2003 91 39 45 10.1016/S0090-8258(03)00461-X 14529660

[B27] Ahmed RS Kim RY Duan J Meleth S De Los Santos JF Fiveash JB IMRT dose escalation for positive para-aortic lymph nodes in patients with locally advanced cervical cancer while reducing dose to bone marrow and other organs at risk Int J Radiat Oncol Biol Phys 2004 60 505 512 10.1016/j.ijrobp.2004.03.035 15380585

[B28] Salama JK Roeske JC Mehta N Mundt AJ Intensity-modulated radiation therapy in gynecologic malignancies Curr Treat Options Oncol 2004 5 97 108 10.1007/s11864-004-0042-2 14990204

[B29] D'Souza WD Ahamad AA Iyer RB Salehpour MR Jhingran A Eifel PJ Feasibility of dose escalation using intensity-modulated radiotherapy in posthysterectomy cervical carcinoma Int J Radiat Oncol Biol Phys 2005 61 1062 1070 10.1016/j.ijrobp.2004.07.721 15752885

[B30] Mell LK Tiryaki H Ahn KH Mundt AJ Roeske JC Aydogan B Dosimetric comparison of bone marrow-sparing intensity-modulated radiotherapy versus conventional techniques for treatment of cervical cancer Int J Radiat Oncol Biol Phys 2008 71 1504 1510 10.1016/j.ijrobp.2008.04.046 18640499

[B31] Igdem S Ercan T Alco G Zengin F Ozgules R Geceer G Okkan S Ober A Turkan S Dosimetric comparison of intensity modulated pelvic radiotherapy with 3D conformal radiotherapy in patients with gynecologic malignancies Eur J Gynaecol Oncol 2009 30 547 551 19899413

[B32] http://www.nccn.org/international/international_adaptations.asp

[B33] Koh WJ Controversies in the radiotherapeutic management of cervical cancer J Clin Oncol 2003 21 218s 223s 10.1200/JCO.2003.01.224 12743138

[B34] Begg CB Mazumdar M Operating characteristics of a rank correlation test for publication bias Biometrics 1994 50 1088 1101 10.2307/2533446 7786990

[B35] Egger M Davey Smith G Schneider M Minder C Bias in meta-analysis detected by a simple, graphical test BMJ 1997 315 629 634 10.1136/bmj.315.7109.629 9310563 PMC2127453

[B36] Chen MF Tseng CJ Tseng CC Kuo YC Yu CY Chen WC Clinical outcome in posthysterectomy cervical cancer patients treated with concurrent Cisplatin and intensity-modulated pelvic radiotherapy: comparison with conventional radiotherapy Int J Radiat Oncol Biol Phys 2007 67 1438 1444 10.1016/j.ijrobp.2006.11.005 17394944

[B37] Roeske JC Lujan A Rotmensch J Waggoner SE Yamada D Mundt AJ Intensity-modulated whole pelvic radiation therapy in patients with gynecologic malignancies Int J Radiat Oncol Biol Phys 2000 48 1613 1621 10.1016/S0360-3016(00)00771-9 11121668

[B38] Lujan AE Mundt AJ Yamada SD Rotmensch J Roeske JC Intensity-modulated radiotherapy as a means of reducing dose to bone marrow in gynecologic patients receiving whole pelvic radiotherapy Int J Radiat Oncol Biol Phys 2003 57 516 521 10.1016/S0360-3016(03)00521-2 12957265

[B39] Mundt AJ Mell LK Roeske JC Preliminary analysis of chronic gastrointestinal toxicity in gynecology patients treated with intensity-modulated whole pelvic radiation therapy Int J Radiat Oncol Biol Phys 2003 56 1354 1360 10.1016/S0360-3016(03)00325-0 12873680

[B40] Brixey CJ Roeske JC Lujan AE Yamada SD Rotmensch J Mundt AJ Impact of intensity-modulated radiotherapy on acute hematologic toxicity in women with gynecologic malignancies Int J Radiat Oncol Biol Phys 2002 54 1388 1396 10.1016/S0360-3016(02)03801-4 12459361

[B41] Mell LK Kochanski JD Roeske JC Haslam JJ Mehta N Yamada SD Hurteau JA Collins YC Lengyel E Mundt AJ Dosimetric predictors of acute hematologic toxicity in cervical cancer patients treated with concurrent cisplatin and intensity-modulated pelvic radiotherapy Int J Radiat Oncol Biol Phys 2006 66 1356 1365 10.1016/j.ijrobp.2006.03.018 16757127

[B42] Beriwal S Gan GN Heron DE Selvaraj RN Kim H Lalonde R Kelley JL Edwards RP Early clinical outcome with concurrent chemotherapy and extended-field, intensity-modulated radiotherapy for cervical cancer Int J Radiat Oncol Biol Phys 2007 68 166 171 10.1016/j.ijrobp.2006.12.023 17321070

[B43] Hasselle MD Rose BS Kochanski JD Nath SK Bafana R Yashar CM Hasan Y Roeske JC Mundt AJ Mell LK Clinical outcomes of intensity-modulated pelvic radiation therapy for carcinoma of the cervix Int J Radiat Oncol Biol Phys 2011 80 5 1436 1445 10.1016/j.ijrobp.2010.04.041 20708346

[B44] Salama JK Mundt AJ Roeske J Mehta N Preliminary outcome and toxicity report of extended-field, intensity-modulated radiation therapy for gynecologic malignancies Int J Radiat Oncol Biol Phys 2006 65 1170 1176 10.1016/j.ijrobp.2006.02.041 16730136

[B45] Georg D Georg P Hillbrand M Potter R Mock U Assessment of improved organ at risk sparing for advanced cervix carcinoma utilizing precision radiotherapy techniques Strahlenther Onkol 2008 184 586 591 10.1007/s00066-008-1872-9 19016017

[B46] Mundt AJ Roeske JC Lujan AE Yamada SD Waggoner SE Fleming G Rotmensch J Initial clinical experience with intensity-modulated whole-pelvis radiation therapy in women with gynecologic malignancies Gynecol Oncol 2001 82 456 463 10.1006/gyno.2001.6250 11520140

[B47] Roeske JC Bonta D Mell LK Lujan AE Mundt AJ A dosimetric analysis of acute gastrointestinal toxicity in women receiving intensity-modulated whole-pelvic radiation therapy Radiother Oncol 2003 69 201 207 10.1016/j.radonc.2003.05.001 14643959

[B48] Rose BS Aydogan B Liang Y Yeginer M Hasselle MD Dandekar V Bafana R Yashar CM Mundt AJ Roeske JC Mell LK Normal Tissue Complication Probability Modeling of Acute Hematologic Toxicity in Cervical Cancer Patients Treated with Chemoradiotherapy Int J Radiat Oncol Biol Phys 2011 79 3 800 807 10.1016/j.ijrobp.2009.11.010 20400238 PMC2907446

